# Synthesis, Antimycobacterial Activity and *In Vitro* Cytotoxicity of 5-Chloro-*N*-phenylpyrazine-2-carboxamides

**DOI:** 10.3390/molecules181214807

**Published:** 2013-12-02

**Authors:** Jan Zitko, Barbora Servusová, Pavla Paterová, Jana Mandíková, Vladimír Kubíček, Radim Kučera, Veronika Hrabcová, Jiří Kuneš, Ondřej Soukup, Martin Doležal

**Affiliations:** 1 Faculty of Pharmacy in Hradec Králové, Charles University in Prague, Heyrovského 1203, Hradec Králové 500 05, Czech Republic; E-Mails: barbora.servusova@faf.cuni.cz (B.S.); jana.mandikova@faf.cuni.cz (J.M.); kubicek@faf.cuni.cz (V.K.); kucera@faf.cuni.cz (R.K.); kunes@faf.cuni.cz (J.K.); dolezalm@faf.cuni.cz (M.D.); 2 Department of Clinical Microbiology, University Hospital, Sokolská 581, Hradec Králové 500 05, Czech Republic; E-Mail: pavla.paterova@fnhk.cz; 3 Biomedical Research Center, Sokolská 581, Hradec Králové 500 05, Czech Republic; E-Mails: veronika.hrabcova@fnhk.cz (V.H.); ondrej.soukup@fnhk.cz (O.S.); 4 Department of Chemistry, Faculty of Science, University of Hradec Králové, Jana Koziny 1237, Hradec Králové 500 05, Czech Republic

**Keywords:** pyrazinamide, 5-chloropyrazinamide, anilides, antimycobacterial activity, cytotoxicity

## Abstract

5-Chloropyrazinamide (5-Cl-PZA) is an inhibitor of mycobacterial fatty acid synthase I with a broad spectrum of antimycobacterial activity *in vitro*. Some *N-*phenylpyrazine-2-carboxamides with different substituents on both the pyrazine and phenyl core possess significant *in vitro* activity against *Mycobacterium tuberculosis.* To test the activity of structures combining both the 5-Cl-PZA and anilide motifs a series of thirty 5-chloro-*N*-phenylpyrazine-2-carboxamides with various substituents R on the phenyl ring were synthesized and screened against *M. tuberculosis* H37Rv, *M. kansasii* and two strains of *M. avium*. Most of the compounds exerted activity against *M. tuberculosis* H37Rv in the range of MIC = 1.56–6.25 µg/mL and only three derivatives were inactive. The phenyl part of the molecule tolerated many different substituents while maintaining the activity. *In vitro* cytotoxicity was decreased in compounds with hydroxyl substituents, preferably combined with other hydrophilic substituents. 5-Chloro-*N*-(5-chloro-2-hydroxyphenyl)pyrazine-2-carboxamide (**21**) inhibited all of the tested strains (MIC = 1.56 µg/mL for *M. tuberculosis*; 12.5 µg/mL for other strains). 4-(5-Chloropyrazine-2-carboxamido)-2-hydroxybenzoic acid (**30**) preserved good activity (MIC = 3.13 µg/mL *M. tuberculosis*) and was rated as non-toxic in two *in vitro* models (Chinese hamster ovary and renal cell adenocarcinoma cell lines; SI = 47 and 35, respectively).

## 1. Introduction

Although both relative and absolute incidence of tuberculosis (TB) have been decreasing globally since approximately the beginning of the millennium, tuberculosis remains a serious threat to public health and is the second leading cause of death from infectious diseases. According to the WHO Global Tuberculosis Report 2013 estimates 8.6 million of people developed active form of TB in 2012 and 1.3 million died from the disease (including 320,000 deaths among HIV-positive people) [1]. Besides the HIV co-infection, TB control policy is endangered mainly by increasing resistance to current clinically used antitubercular drugs. In 2012, there were 450,000 new cases of multidrug resistant tuberculosis (MDR-TB) and 170,000 deaths from MDR-TB [1]. Therefore, there is still a need for the development of new antitubercular medicines, especially those active against resistant strains of mycobacteria.

5-Chloropyrazine-2-carboxamide (5-Cl-PZA) was previously shown to possess *in vitro* antimycobacterial activity, not only against *M. tuberculosis* (*M. tbc*), but also against pyrazinamide (PZA)-resistant strains and against atypical mycobacteria naturally resistant to PZA—*M. kansasii*, *M. smegmatis*, *M. fortuitum*, *M. avium* [2]. The Fatty Acid Synthase I (FAS I) system of mycobacteria was proposed to be the possible target of 5-Cl-PZA based on the observation that overexpression of *fas1* gene in *M. smegmatis* conferred resistance to 5-Cl-PZA [3]. Indeed, later studies confirmed that 5-Cl-PZA inhibited the FAS I in both whole cell [4] and isolated enzyme [4,5] assays. Sayahi *et al*. showed by Saturation Difference Transfer NMR experiments that 5-Cl-PZA binds competitively to the NADPH binding site of FAS I and that the affinity of 5-Cl-PZA is superior to that of non-substituted PZA [6]. Little is known about the possible *in vivo* activity of 5-Cl-PZA. A recent study using a chronic murine TB-model failed to confirm the *in vivo* activity of 5-Cl-PZA [7]. The possible explanation could be its *in vivo* metabolic instability [8] and/or poor pharmacokinetics. If so, these issues might be solved by proper structural modifications of 5-Cl-PZA.

Some *N*-phenylpyrazine-2-carboxamides, *i.e.*, anilides of pyrazine-2-carboxylic acid (POA), with various substituents both on the pyrazine and phenyl core were previously described to possess significant antimycobacterial activity [9,10]. The published compounds were anilides of non-substituted pyrazine-2-carboxylic acid, 6-chloro-POA, 5-*tert*-butyl-POA; and 5-*tert*-butyl-6-chloro-POA. Based on the facts describe above, we decided to synthesize and probe the potential antimycobacterial activity of 5-chloro-*N*-phenylpyrazine-2-carboxamides, *i.e.*, compounds combining the 5-Cl-PZA motif possessing FAS I inhibiting activity and the anilide motif. The preliminary data of first six compounds from this series were published recently [11] and showed a significant level of antimycobacterial activity in low micromolar concentration against *M. tuberculosis* H37Rv. This paper presents the extended study of 5-chloro-*N*-phenylpyrazine-2-carboxamides, their antimycobacterial activity against four different mycobacterial strains and *in vitro* cytotoxicity of the compounds with significant activity. The basic insights into the structure-activity and structure-toxicity relationships of these compounds is presented too.

## 2. Results and Discussion

### 2.1. Chemistry

**Scheme 1:**
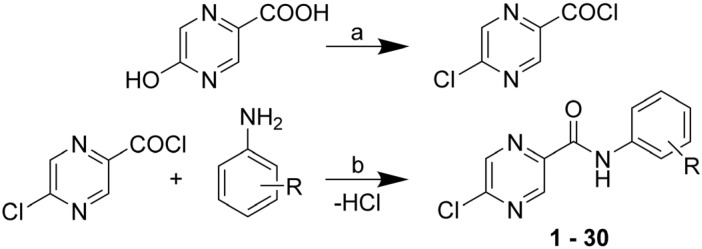
Synthesis of the title compounds.

The title 5-chloro-*N*-phenylpyrazine-2-carboxamide compounds **1**–**30** were synthesized from commercially available 5-hydroxypyrazine-2-carobxylic acid (5-OH-POA). The formation of the corresponding acyl chloride was simultaneously accompanied with the substitution of 5-OH group with chlorine. For reagents and conditions see Scheme 1. The overall yields of the two-step reaction ranged from 18% to 89% (of chromatographically pure product). Final products were isolated as white, beige, pale yellow or yellow solids.

*Reagents and conditions*: (a) SOCl_2_, DMF, anhydrous PhMe, 100 °C, 1 h; (b) TEA, anhydrous acetone, RT.

The compounds were characterized by ^1^H-NMR, ^13^C-NMR, FT-IR spectroscopy, melting point and elemental analysis. The analytical data were fully consistent with proposed structures. The results of the elemental analyses were in the range of ±0.3% relative to calculated values. The parent compound **1** (with a non-substituted benzene ring) was also analysed by ion-trap MS. The full-scan spectrum contained the [M+H]^+^ ion at *m*/*z* 234. The loss of water was observed in the MS2 spectrum and ion at *m*/*z* 216 was found. The further fragmentation of ion at *m*/*z* 216 produced the main fragments corresponding to: (a) the loss of HCl (*m*/*z* 180), and (b) the benzene ring (*m*/*z* 77). NMR and MS spectra of compound **1** (internal laboratory code JZ-90) are included in the Supplementary Material.

### 2.2. Lipophilicity

**Figure 1:**
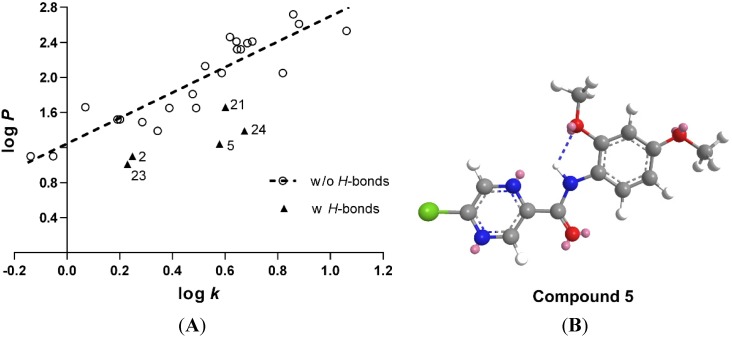
(**A**) Correlation of predicted lipophilicity parameter log*P* and experimentally determined log*k.* Linear regression parameters: *R*^2^ = 0.823, *s* = 0.2142, *F* = 93.03, *n* = 22. Compounds capable of *H-*bonds formation by their *ortho* substituents on the phenyl ring are indicated by triangle marks and were omitted from regression analysis. (**B**) Model of compound **5** including the intramolecular *H-*bond.

Lipophilicity is one of the most important physico-chemical properties determining the biological activity of small molecules, affecting the non-specific diffusion through biological membranes. It is well known that antimycobacterial activity is often enhanced by increased lipophilicity, which facilitates the penetration through highly lipophilic mycobacterial outer envelope and cell wall. The lipophilicity of the prepared compounds was predicted as log*P* using commercially available software CS ChemDraw Ultra ver. 12.0 (CambridgeSoft, Cambridge, MA, USA). Additionally, the lipophilicity was measured experimentally by RP-HPLC and expressed as log*k* derived from retention times of individual compounds (see Table 1). The plot of computer predicted log*P vs.* measured log*k* (Figure 1) indicated a linear correlation, although a few of the compounds were apparently below the expected line of regression, indicating that the computational algorithm underestimated their lipophilicity. Most of these mispredicted compounds had an *ortho* substituent capable of *H-*bond formation (2-OH, 2-OCH_3_, 2-NO_2_ in compounds **2**, **5**, **21**, **23**, and **24**). Chem3D Pro’s (CambridgeSoft) built-in MM2 energy minimization function with default parameters was used to study the possibility of intramolecular *H-*bond formation. Indeed, all of these compounds were capable of forming the *H-*bond between the *ortho* substituent on the phenyl ring (lone electron pair as acceptor) and the hydrogen of the amide function (donor). The amide bond was in the *trans* configuration. See the model of compound **5** in Figure 1B for a representative example. As found in literature, the existence of this *H-*bond was confirmed experimentally for some similar anilides of POA (e.g., with *ortho-*NO_2_ substituent) [12].

### 2.3. Biological Activity

#### 2.3.1. *In Vitro* Antimycobacterial Activity

The prepared compounds were screened for *in vitro* whole cell antimycobacterial activity against *M. tbc* H37Rv, *M. kansasii* and two strains of *M. avium* using a micro-plate dilution method [13]. Firstly, the compounds were tested in concentrations 100–50–25–12.5–6.25–3.13–1.56 µg/mL. Based on the results selected compounds with MIC ≤ 3.13 µg/mL were retested using extended dilution scale up to 0.39 µg/mL (Table 1, values in parentheses). The differences between MIC values obtained in this retest and original values were less or equal to two steps on the dilution scale, which is a usual error for this type of assay. The MIC values detected for standard 5-chloropyrazine-2-carboxamide (5-Cl-PZA; see Table 1) were in good agreement with values reported in literature (MIC = 8–32 μg/mL for PZA-sensitive *M. tbc* strains, MIC = 8–64 μg/mL for PZA-resistant strains) [2].

**Table 1:** Summary of prepared compounds, their antimycobacterial activity expressed as MIC (μg/mL), HepG2 cytotoxicity, and calculated and measured lipophilicity parameters ^a^.

Compound		Antimycobacterial activity MIC (μg/mL)				Cytotoxicity		Lipophilicity	
No.	R	*M. tbc* ^b^	*M.kansasii* ^b^	*M.avium* ^b^	*M. avium* ^b^	IC_50_ (μM)	SI ^c^	log*P*	log*k*
**1**	H	3.13 (1.56)	25	>100	50	2.55	0.19	1.49	0.286
**2**	2-OH	3.13 (0.78)	n.d.	>100	>100	30.00	2.39	1.10	0.248
**3**	3-OH	6.25	50	>100	100	32.10	1.28	1.10	−0.053
**4**	4-OH	3.13 (12.5)	100	>100	50	68.60	5.47	1.10	−0.138
**5**	2,4-(OCH_3_)_2_	>50	>50	>50	>50	n.a.	n.a.	1.24	0.579
**6**	2,5-(CH_3_)_2_	1.56 (1.56)	>100	>100	>100	11.66	1.96	2.46	0.618
**7**	4-C_2_H_5_	1.56 (0.78)	n.d.	>100	>100	7.17	2.41	2.39	0.685
**8**	4-*i*-Pr	1.56	n.d.	>100	>100	14.44	2.55	2.72	0.859
**9**	2-F	6.25	12.5	>100	>100	n.a.	n.a.	1.65	0.490
**10**	3-F	6.25	12.5	>100	>100	n.a.	n.a.	1.65	0.389
**11**	2,4-F_2_	3.13	6.25	>50	>50	n.a.	n.a.	1.81	0.477
**12**	2-Cl	3.13 (0.78)	n.d.	>100	>100	6.72	0.58	2.05	0.819
**13**	3-Cl	6.25 (3.13)	25	25	25	n.a.	n.a.	2.05	0.587
**14**	3,4-Cl_2_	3.13	>100	>100	>100	9.10	0.88	2.61	0.881
**15**	2,4,5-Cl_3_	>100	>100	>100	>100	n.a.	n.a.	3.16	n.a.
**16**	3-Br	25	25	>100	>100	n.a.	n.a.	2.32	0.646
**17**	4-Br	3.13	6.25	>100	>100	n.a.	n.a.	2.32	0.660
**18**	2-Cl-4-I	12.5	1.56	>50	>50	n.a.	n.a.	3.41	n.a.
**19**	2-CH_3_-5-F	3.13 (6.25)	25	>100	>100	n.a.	n.a.	2.13	0.525
**20**	2-Cl-5-CH_3_	1.56	25	>100	>100	15.84	2.86	2.53	1.061
**21**	5-Cl-2-OH	1.56 (6.25)	12.5	12.5	12.5	40.59	7.39	1.66	0.602
**22**	3-Cl-4-OH	3.13 (0.39)	>100	50	25	12.9	1.17	1.66	0.070
**23**	2-OH-5-NO_2_	1.56 (1.56)	n.d.	50	50	1.52	0.29	1.01	0.230
**24**	2-NO_2_	12.5	n.d.	>100	>100	n.a.	n.a.	1.39	0.674
**25**	3-NO_2_	3.13	n.d.	>100	>100	32.70	2.91	1.39	0.344
**26**	3-CN	25	3.13	>50	>50	n.a.	n.a.	1.52	0.192
**27**	4-CN	>100	>100	>100	>100	n.a.	n.a.	1.52	0.201
**28**	3-CF_3_	3.13 (6.25)	12.5	>100	>100	41.39	3.99	2.41	0.643
**29**	4-CF_3_	1.56 (3.13)	12.5	>100	>100	8.50	1.64	2.41	0.704
**30**	4-COOH-3-OH	3.13	n.d.	>100	>100	n.a.	n.a.	0.66	n.a.
**5-Cl-PZA**	−	25	12.5	>100	>100	1594	10.0	−0.41	n.a.
**PZA**	−	6.25–12.5	>100	>100	>100	>10^4^	>196	−1.31	−0.687
**INH**	−	0.39–0.78	12.5–25	12.5–25	3.13–6.25	79 × 10^3 d^	n.a.	−0.64	−0.743

^a^ Data in parentheses represent the MIC values in confirmation retest; n.d.—not detected due to decreased viability of the strain, data not reproducible; n.a.—not available; 5-Cl-PZA—5-chloropyrazine-2-carboxamide; PZA—pyrazinamide; INH—isoniazid; ^b^ Tested strains from left to right *M. tuberculosis* H37Rv, *M. kansasii* Hauduroy CNCTC My 235/80, *M. avium* ssp*. avium* Chester CNCTC My 80/72, *M. avium* CNCTC My 152/73; ^c^ SI values calculated for *M. tbc* as IC_50_/MIC (in μM) using the lower MIC values; ^d^ Data from literature [14] in a comparable HepG2 cytotoxicity assay: PZA—IC_50_ = 79.1 mM, INH—IC_50_ = 78.8 mM.

As seen in Table 1, most of the compounds exerted antimycobacterial activity against *M. tbc* H37Rv in the range of MIC = 1.56–6.25 µg/mL. The aniline part of the molecule tolerated many different substituents R while maintaining the activity—both electron-donating (-OH, alkyl substituents) and electron-withdrawing substituents (3-NO_2_, 3-CF_3_, 4-CF_3_). All compounds with simple alkyl substituent R (**6**–**8**) exerted MIC = 1.56 µg/mL or lower (*M. tbc* H37Rv). The combination of halogen substituent with methyl (compounds **19**, **20**), hydroxyl (**21**, **22**) or nitro substituent (**23**) seemed to be advantageous and produced compounds with MIC = 3.13 µg/mL or lower. Compounds with R = CN were inactive (**27**) or of low activity (**26**).

The lipophilicity (expressed as log*k*) did not correlate with antimycobacterial activity. However, highly lipophilic compounds with multiple halogen substituents (compounds **15**, **18**) suffered from low solubility in testing medium and were inactive or weakly active. Insufficient solubility in testing medium was observed also with inactive compound **5** (2,4-dimethoxy derivative), despite of its rather low lipophilicity.

The activity against *M. kansasii* was generally lower in comparison to the activity against *M. tbc* H37Rv. According to incomplete results, the fluorinated (**9**–**11**) and brominated (**16**, **17**) compounds preserved the same or similar level of MIC against *M. kansasii* and *M. tbc* H37Rv. With the exception of 3-CN derivative **26**, compounds with significant activity against *M. kansasii* (MIC ≤ 6.25 µg/mL) had a halogen substituent R. The most lipophilic (log*P*) compound **18** with two halogen substituents on the phenyl ring (R = 2-Cl-4-I) was the most active against *M. kansasii*. On the contrary hydrophilic substituents R (hydroxyl in **3** and **4**) lead to inactive derivatives. We suggest that in this series halogenation on the phenyl ring and increased lipophilicity are advantageous with the respect to activity against *M. kansasii.*

Only three of the tested compounds (**13**, **21**, **22**) were active against *M. avium* strains (weak activity, MIC = 12.5–25 µg/mL). Interestingly, all of them had chlorine substitution in *meta* position of the phenyl ring. This could indicate a steric need for a large (and hydrophobic) substituent in this position.

Doležal *et al*. published several papers on synthesis and antimycobacterial activity of substituted anilides of POA, 6-Cl-POA, 5-*tert-*Bu-POA, and 5-*tert-*Bu-6-Cl-POA. The summary of structure-activity relationships within these series was published recently [9,10], including the references to original articles. The antimycobacterial activity (*M. tbc* H37Rv) was indicated as percent of growth inhibition at fixed concentration of 6.25 µg/mL. Only six out of 91 anilides exerted the inhibition of 80% or higher and 21 compounds were completely inactive [10]. The best reported MIC values (measured only for compounds with inhibition over 90%) were from 3.13 to 12.5 µg/mL [10]. Judged from the relatively large portion of inactive anilides from the previous series (21/91) compared with the number of inactive anilides of the title series of *N*-phenyl-5-chloropyrazine-2-carboxamides (three out of 30), we conclude that the 5-chloro substitution of the pyrazine nucleus is the most advantageous from all of the discussed series. This is supported by another study which found only moderate to weak activity (MIC = 50–100 µg/mL) for some anilides of non-substituted POA [15].

Recently we have published a letter [11] on the antimycobacterial activity of *N*-benzyl-5-chloropyrazine-2-carboxamides, *i.e.*, the methylene homologues of the anilides discussed herein. The direct comparison of anilides **5**, **9**, **12**, **13**, **17**, and **25** with the respective *N-*benzyl derivatives with identical substitution patterns on the benzene ring clearly reveals that all of the compared anilides possess significantly better activity against *M. tbc* H37Rv. Similarly, anilides **1** and **28** had better activity compared with their *N-*benzyl homologues presented in another publication [16]. Generally, the MIC values for *N*-benzyl-5-chloropyrazine-2-carboxamides ranged from 12.5 to 25 µg/mL [11,16], whereas 20 of 30 anilides of 5-Cl-POA discussed in this article reached MIC ≤ 3.13 µg/mL (in primary or repeated testing). As all of the compounds discussed in this paragraph were tested by the same methodology and by the same researcher, the comparison is of a significant value. We conclude that antimycobacterial activity of *N*-phenyl-5-chloropyrazine-2-carboxamides is superior to the activity of *N*-benzyl-5-chloropyrazine-2-carboxamides, *i.e.*, that incorporation of the -CH_2_– bridge leads to significant decrease of antimycobacterial activity.

#### 2.3.2. *In Vitro* Cytotoxicity

Drug-induced hepatotoxicity is a common side-effect of many of the clinically used antitubercular agents (PZA, INH, rifampicin) [17]. Tuberculosis treatment regimens are always multi-drug; therefore, newly introduced antitubercular agent would be probably used in a combination with at least some of the classic antituberculars with significant hepatotoxicity. Thus the hepatotoxicity of new compounds being developed as potential antituberculars should be considered very carefully.

To evaluate the potential hepatotoxicity, the IC_50_ of selected title compounds with promising antitubercular activity were determined in a hepatocellular carcinoma cell line (HepG2) *in vitro* model. This model has been widely used to study the hepatotoxicity of various antitubercular drugs before [18,19]. The decrease of viability of HepG2 cells was measured using a standard protocol [20] based on colorimetric method measuring reduction of tetrazolium salt.

The tested compounds exerted significant hepatotoxicity with IC_50_ values from units to tens of µM. With the exception of compound **23** where combined with aromatic nitro group, the presence of hydroxyl on the phenyl ring lead to relative decrease of hepatotoxicity, as in compounds **2**–**4**, and **21**. Four of six compounds with the highest IC_50_ values (lowest toxicity) bore a hydroxyl. The -OH group is probably a conjugation site involved in the drug metabolism - detoxification process.

The obtained IC_50_ values were used to calculate the selectivity indexes (SI) related to antimycobacterial activity against *M. tbc* H37Rv. None of the compounds had SI > 10, which is a limit considered safe for further development. Promising SI values (SI > 5) were obtained for compounds **4** and **21**, both of them with R = OH.

To evaluate the *in vitro* cytotoxicity more comprehensively, we chose the afforementioned compunds **4** and **21** with the lowest IC_50_ values in HepG2 model, together with compound **30** (R = 4-COOH 3-OH), which we presumed could also possess diminished cytotoxicity because of its low hydrophobicity and hydroxyl substitution, and assessed them for *in vitro* cytotoxicity on renal cell adenocarcinoma (ACHN) and Chinese hamster ovary (CHO-K1) cell lines. Table 2 presents the comparison of IC_50_ values for HepG2, CHO-K1 and ACHN cell lines accompanied by antimycobacterial activity of individual compounds against *M. tuberculosis* H37Rv (MIC converted to molar concentrations). The level of cytotoxicity was similar among all three cell lines, for compouds **4** and **21** the IC_50_ was at tens of µM, leading to SI = 3.8–9.3. Notably, compound **30** can be designated as non-toxic for both CHO-K1 and ACHN cell lines with IC_50_ at hundreds of µM. Selectivity indexes of compund **30** are of value which is favourable for furhter development. This finding confirms that hydroxyl substitution of the phenyl ring, preferrably combined with other lipophilicity decreasing substituents, leads to less toxic or non-toxic derivatives in the series of 5-chloro-*N-*phenylpyrazine-2-carboxamides.

**Table 2:** Cytotoxic effect of selected compounds on different cell lines expressed as IC_50_ and SI.

Compound	*M. tbc* H37Rv	HepG2		CHO-K1		ACHN	
	MIC (µM)	IC_50_ (µM)	SI	IC_50_ (µM)	SI	IC_50_ (µM)	SI
**4**	12.5	69	5.5	48 ± 4	3.8	100 ± 39	8.0
**21**	5.5	41	7.4	39 ± 2	7.1	51 ± 14	9.3
**30**	10.7	n.a.	n.a.	502 ± 122	47.1	371 ± 96	34.8
**5-Cl PZA**	158.7	1594	10.0	290 ± 43	0.9	540 ± 120	1.7

Values are expressed as the IC_50_: Mean ± SEM (µM) (n = 3) where applicable. SI = IC_50_/MIC.

#### 2.3.3. *In Vitro* Antibacterial and Antifungal Activity

As a complementary screening test, all of the final compounds were tested for activity against selected pathogenic bacterial and fungal species, but no significant activity compared with standards was detected.

## 3. Experimental

### 3.1. General

All chemicals (unless stated otherwise) were purchased from Sigma-Aldrich (Schnelldorf, Germany). The reaction process and the purity of final compounds were checked using Merck Silica 60 F254 TLC plates (Merck, Darmstadt, Germany). Flash chromatography of the final compounds was run on automated chromatograph CombiFlash Rf (Teledyne Isco, Lincoln, NE, USA) using columns filled with Kieselgel 60, 0.040–0.063 mm (Merck), detection wavelength 280 nm. NMR spectra were recorded on Varian VNMR S500 (Varian, Palo Alto, CA, USA) at 500 MHz for ^1^H and 125 MHz for ^13^C or at Varian Mercury VX-BB 300 at 300 MHz for ^1^H and 75 MHz for ^13^C. The spectra were recorded in DMSO-*d_6_* or CDCl_3_ at ambient temperature. The chemical shifts as δ values in ppm are indirectly referenced to tetramethylsilane (TMS) via the solvent signal. IR spectra were recorded on Nicolet Impact 400 (Nicolet, Madison, WI, USA) using ATR Ge method. Elemental analysis was performed on CE Instruments EA-1110 CHN analyser (CE Instruments, Wigan, UK). All values are given as percentages. Melting points were determined in open capillary on Stuart SMP30 melting point apparatus (Bibby Scientific Limited, Staffordshire, UK) and are uncorrected. The mass spectra were recorded in the mixture of MeOH, water, formic acid (80:20:0.01 v/v/v) using LCQ Advantage Max ion-trap mass spectrometer (Thermo Finnigan, San Jose, CA, USA). The sample was ionised using APCI probe in a positive mode. Yields are given as percentages and refer to the amount of chromatographically pure product after all purification steps.

### 3.2. Synthesis and Purification of Final Compounds

*General procedure*: 5-Hydroxypyrazine-2-carboxylic acid (5-OH-POA, 300 mg, 2.14 mmol) was dispersed in dry toluene (approx. 30 mL). Thionyl chloride (SOCl_2_, approximately 1.0 mL, 14 mmol) was added to the reaction mixture, followed by a catalytic amount (1–2 drops) of *N*,*N*-dimethylformamide (DMF). The reaction mixture was heated and stirred in an oil bath under a condenser at 100 °C for approx. 1 h. During the course of reaction the starting solid 5-OH-POA dissolved (chemically changed) and the reaction mixture turned brown-red. When no further conversion of the solid could be observed (usually there was a small amount of dark solid particles left), the solvents were decanted from the dark residue and concentrated *in vacuo*. To remove the unreacted SOCl_2_, the residue was azeotroped with dry toluene (3 × 20 mL). The crude 5-chloropyrazine-2-carbonyl chloride product , obtained in the form of brown-red viscous liquid, was diluted with dry acetone (10 mL) and added dropwise to the stirred solution of respective aniline (1.71 mmol, 0.8 molar equivalents) and triethylamine (433 mg, 4.18 mmol, 2 molar equivalents) in dry acetone (20 mL). The product precipitated from the reaction mixture. The mixture was stirred at laboratory temperature for 30 min and the completeness was checked by TLC (silica 60 F_254_, hexane–EtOAc 3:1). The reaction mixture was adsorbed to silica by removing the solvents *in vacuo* and the product was purified by flash chromatography (silica, 0%–25% EtOAc in hexane gradient elution) and recrystallized from EtOH/H_2_0 if needed. *Note*: For compounds with higher polarity, e.g., compounds **2**–**4** and **30**, it was necessary to increase the strength of the mobile phase for flash chromatography. Usually gradient elution 0%–60% EtOAc in hexane was sufficient, although for compound **30** we had to use EtOAc with 10% of MeOH.

### 3.3. Data of the Prepared Target Compounds

Analytical data of compounds **5**, **9**, **12**, **13**, **17**, and **25** were published previously in a preliminary study [11].

*5-Chloro-N-phenylpyrazine-2-carboxamide* (**1**). White solid. Yield: 43%. mp 157.2–158.1 °C. ^1^H-NMR (300 MHz, CDCl_3_) δ 9.48 (1H, bs, NH), 9.27 (1H, d, *J* = 1.4 Hz, H3), 8.57 (1H, d, *J* = 1.4 Hz, H6), 7.76–7.70 (2H, m, H2', H6'), 7.44–7.36 (2H, m, H3', H5'), 7.22–7.15 (1H, m, H4'). ^13^C-NMR (75 MHz, CDCl_3_) δ 159.7, 152.3, 144.2, 142.6, 142.3, 137.0, 129.2, 125.0, 119.8. IR (ATR Ge, cm^−1^): 3323 (NH, CONH), 2357, 1667 (C=O, CONH), 1598, 1536, 1444, 1310, 1137, 1115, 1024, 897, 754, 694. Anal. Calcd. for C_11_H_8_Cl_1_N_3_O_1_ (MW 233.65): C, 56.55; H, 3.45; N, 17.98. Found: C, 56.38; H, 3.57; N, 18.05.

*5-Chloro-N-(2-hydroxyphenyl)pyrazine-2-carboxamide* (**2**). Pale yellow solid. Yield: 46%. mp 220.9–221.6 °C. ^1^H-NMR (300 MHz, DMSO-*d_6_*) δ 10.35 (1H, s, OH), 10.07 (1H, bs, NH), 9.13–9.11 (1H, m, H3), 8.95–8.93 (1H, m, H6), 8.28 (1H, dd, *J* = 8.0 Hz, *J* = 1.4 Hz, H3'), 7.02–6.81 (3H, m, H4', H5', H6').^13^C-NMR (75 MHz, DMSO-*d_6_*) δ 159.5, 151.4, 146.9, 143.6, 143.4, 143.1, 125.8, 124.9, 119.6, 119.5, 115.0. IR (ATR Ge, cm^−1^): 3319 (NH, CONH), 3090, 1655 (C=O, CONH), 1551, 1454, 1373, 1314, 1284, 1241, 1150, 1116, 1020, 907, 856, 758, 699. Anal. Calcd. for C_11_H_8_Cl_1_N_3_O_2_ (MW 249.65): C, 52.92; H, 3.23; N, 16.83. Found: C, 53.01; H, 3.40; N, 16.98.

*5-Chloro-N-(3-hydroxyphenyl)pyrazine-2-carboxamide* (**3**). Pale yellow solid. Yield: 39%. mp 225.1–226.3 °C. ^1^H-NMR (300 MHz, DMSO-*d_6_*) δ 10.63 (1H, bs, NH), 9.53 (1H, s, OH), 9.16 (1H, d, *J* = 1.4 Hz, H3), 8.98 (1H, d, *J* = 1.4 Hz, H6), 7.50 (1H, t, *J* = 2.1 Hz, H2'), 7.34–7.28 (1H, m, H4'), 7.19 (1H, t, *J* = 8.1 Hz, H5'), 6.65–6.57 (1H, m, H6'). ^13^C-NMR (75 MHz, DMSO-*d_6_*) δ 160.9, 157.7, 151.0, 144.2, 144.2, 143.1, 139.2, 129.5, 111.7, 111.6, 107.8. IR (ATR Ge, cm^−1^): 3318 (NH, CONH), 3268, 1682, 1666 (C=O, CONH), 1615, 1544, 1451, 1279, 1196, 1137, 1116, 1022, 895, 784, 685. Anal. Calcd. for C_11_H_8_Cl_1_N_3_O_2_ (MW 249.65): C, 52.92; H, 3.23; N, 16.83. Found: C, 53.13; H, 3.22; N, 16.72.

*5-Chloro-N-(4-hydroxyphenyl)pyrazine-2-carboxamide* (**4**). Yellow solid. Yield: 45%. mp 204.8–206.7 °C. ^1^H-NMR (300 MHz, DMSO-*d_6_*) δ 10.49 (1H, bs, NH), 9.32 (1H, s, OH), 9.06 (1H, d, *J* = 1.4 Hz, H3), 8.87 (1H, d, *J* = 1.4 Hz, H6), 7.67–7.58 (2H, m, AA', BB', H2', H6'), 6.77–6.69 (2H, m, AA', BB', H3', H5'). ^13^C-NMR (75 MHz, DMSO-*d_6_*) δ 160.4, 154.4, 150.9, 144.3, 144.0, 143.0, 129.8, 122.5, 115.2. IR (ATR Ge, cm^−1^): 3339 (NH, CONH), 3291, 1689 (C=O, CONH), 1639, 1602, 1554, 1510, 1444, 1264, 1219, 1142, 1115, 1026, 901, 836, 809, 668. Anal. Calcd. for C_11_H_8_Cl_1_N_3_O_2_ (MW 249.65): C, 52.92; H, 3.23; N, 16.83. Found: C, 53.07; H, 3.11; N, 16.57.

*5-Chloro-N-(2,5-dimethylphenyl)pyrazine-2-carboxamide* (**6**). White solid. Yield: 77%. mp 139.7–140.5 °C. ^1^H-NMR (300 MHz, CDCl_3_) δ 9.46 (1H, bs, NH), 9.27 (1H, d, *J* = 1.4 Hz, H3), 8.58 (1H, d, *J* = 1.4 Hz, H6), 8.02 (1H, s, H6'), 7.11 (1H, d, *J* = 7.7 Hz, H3'), 6.93 (1H, d, *J* = 7.7 Hz, H4'), 2.37 (3H, s, CH_3_), 2.34 (3H, s, CH_3_). ^13^C-NMR (75 MHz, CDCl_3_) δ 159.6, 152.3, 144.1, 142.9, 142.4, 136.8, 134.8, 130.3, 126.0, 125.0, 122.1, 21.2, 17.1. IR (ATR Ge, cm^−1^): 3370 (NH, CONH), 1697 (C=O, CONH), 1583, 1541, 1450, 1256, 1146, 1127, 1023, 897, 811. Anal. Calcd. for C_13_H_12_Cl_1_N_3_O_1_ (MW 261.71): C, 59.66; H, 4.62; N, 16.06. Found: C, 59.38; H, 4.89; N, 16.13.

*5-Chloro-N-(4-ethylphenyl)pyrazine-2-carboxamide* (**7**). White crystalline. Yield: 51%. mp 155.6–156.5 °C. ^1^H-NMR (300 MHz, CDCl_3_) δ 9.44 (1H, bs, NH), 9.26 (1H, d, *J* = 1.4 Hz, H3), 8.56 (1H, d, *J* = 1.4 Hz, H6), 7.67–7.60 (2H, m, AA', BB', H2', H6'), 7.26–7.19 (2H, m, AA', BB', H3', H5'), 2.65 (2H, q, *J* = 7.7 Hz, CH_2_), 1.24 (3H, t, *J* = 7.7 Hz, CH_3_). ^13^C-NMR (75 MHz, CDCl_3_) δ 159.6, 152.2, 144.2, 142.7, 142.3, 141.2, 134.6, 128.5, 119.9, 28.3, 15.6. IR (ATR Ge, cm^−1^): 3355 (NH, CONH), 2967 (CH_3_), 1673 (C=O, CONH), 1595, 1530, 1518, 1459, 1414, 1311, 1137, 1126, 1024, 901, 834, 660. Anal. Calcd. for C_13_H_12_Cl_1_N_3_O_1_ (MW 261.71): C, 59.66; H, 4.62; N, 16.06. Found: C, 59.70; H, 4.58; N, 15.93.

*5-Chloro-N-(4-isopropylphenyl)pyrazine-2-carboxamide* (**8**). White crystalline. Yield: 44%. mp 151.1–152.8 °C. ^1^H-NMR (300 MHz, CDCl_3_) δ 9.44 (1H, bs, NH), 9.27 (1H, d, *J* = 1.0 Hz, H3), 8.57 (1H, d, *J* = 1.0 Hz, H6), 7.69–7.61 (2H, m, AA', BB', H2', H6'), 7.30–7.22 (2H, m, AA', BB', H3', H5'), 3.01–2.83 (1H, m, CH), 1.26 (6H, d, *J* = 6.9 Hz, CH_3_). ^13^C-NMR (75 MHz, CDCl_3_) δ 159.6, 152.2, 145.8, 144.2, 142.7, 142.3, 134.7, 127.1, 119.9, 33.6, 24.0. IR (ATR Ge, cm^−1^): 3360 (NH, CONH), 2959 (CH3), 2360, 1677 (C=O, CONH), 1594, 1518, 1415, 1310, 1134, 1021, 902, 831, 660. Anal. Calcd. for C_14_H_14_Cl_1_N_3_O_1_ (MW 275.73): C, 60.99; H, 5.12; N, 15.24. Found: C, 61.11; H, 5.27; N, 15.41.

*5-Chloro-N-(3-fluorophenyl)pyrazine-2-carboxamide* (**10**). White to pale yellow crystalline. Yield: 56%. mp 144.5–145.5 °C. ^1^H-NMR (300 MHz, CDCl_3_) δ 9.53 (1H, bs, NH), 9.27 (1H, d, *J* = 1.4 Hz, H3), 8.58 (1H, d, *J* = 1.4 Hz, H6), 7.74–7.67 (1H, m, H2'), 7.42–7.29 (2H, m, H5', H6'), 6.94–6.84 (1H, m, H4'). ^13^C-NMR (75 MHz, CDCl_3_) δ 163.0 (d, *J* = 245.4 Hz), 159.9, 152.6, 144.3, 142.4, 142.2, 138.4 (d, *J* = 10.9 Hz), 130.3 (d, *J* = 9.4 Hz), 115.2 (d, *J* = 3.2 Hz), 111.8 (d, *J* = 21.4 Hz), 107.4 (d, *J* = 26.6 Hz). IR (ATR Ge, cm^−1^): 3364 (NH, CONH), 1679 (C=O, CONH), 1616, 1532, 1442, 1273, 1153, 1132, 1024, 875, 785, 682, 663. Anal. Calcd. for C_11_H_7_Cl_1_F_1_N_3_O_1_ (MW 251.64): C, 52.50; H, 2.80; N, 16.70. Found: C, 52.33; H, 2.84; N, 16.55.

*5-Chloro-N-(2,4-difluorophenyl)pyrazine-2-carboxamide* (**11**). White to pale yellow solid. Yield: 49%. mp 176.6–178.1 °C. ^1^H-NMR (300 MHz, CDCl_3_) δ 9.67 (1H, bs, NH), 9.25 (1H, d, *J* = 1.8 Hz, H3), 8.60 (1H, d, *J* = 1.8 Hz, H6), 8.53–8.40 (1H, m, H3'), 7.00–6.88 (2H, m, H5', H6'). ^13^C-NMR (75 MHz, CDCl_3_) δ 159.8, 159.0 (d, *J* = 247.6 Hz), 158.9 (d, *J* = 247.7 Hz), 154.5 (d, *J* = 12.7 Hz), 152.7, 144.1, 142.6, 142.2, 122.4 (d, *J* = 9.2 Hz), 111.4 (dd, *J* = 21.9 Hz, *J* = 3.5 Hz), 103.9 (dd, *J* = 26.5 Hz, *J* = 23.0 Hz). IR (ATR Ge, cm^−1^): 3362 (NH, CONH), 3057, 1694 (C=O, CONH), 1533, 1428, 1255, 1146, 1138, 1118, 1023, 963, 871, 842, 653. Anal. Calcd. for C_11_H_6_Cl_1_F_2_N_3_O_1_ (MW 269.64): C, 49.00; H, 2.24; N, 15.58. Found: C, 48.94; H, 2.03; N, 15.71.

*5-Chloro-N-(3,4-dichlorophenyl)pyrazine-2-carboxamide* (**14**). White solid. Yield: 87%. mp 185.5–186.9 °C. ^1^H-NMR (500 MHz, CDCl_3_) δ 9.49 (1H, bs, NH), 9.25 (1H, d, *J* = 1.4 Hz, H3), 8.57 (1H, d, *J* = 1.5 Hz, H6), 7.97 (1H, d, *J* = 2.5 Hz, H2'), 7.57 (1H, dd, *J* = 8.7, 2.6 Hz, H6'), 7.44 (1H, d, *J* = 8.7 Hz, H5'). ^13^C-NMR (126 MHz, CDCl_3_) δ 159.87, 152.77, 144.25, 142.43, 141.95, 136.40, 133.08, 130.74, 128.29, 121.49, 119.01. IR (ATR Ge, cm^−1^): 3354 (NH, CONH), 2360, 2342, 1692 (C=O, CONH), 1578, 1520, 1478, 1463, 1458, 1388, 1133, 1024, 919, 882, 824, 668. Anal. Calcd. for C_11_H_6_Cl_3_N_3_O_1_ (MW 302.54): C, 43.67; H, 2.00; N, 13.89. Found: C, 43.81; H, 2.24; N, 14.03.

*5-Chloro-N-(2,4,5-trichlorophenyl)pyrazine-2-carboxamide* (**15**). White solid. Yield: 73%. mp 189.3–192.4 °C. ^1^H-NMR (300 MHz, CDCl_3_) δ 10.13 (1H, bs, NH), 9.25 (1H, d, *J* = 1.2 Hz, H3), 8.82 (1H, s, H3'), 8.63 (1H, d, *J* = 1.2 Hz, H6), 7.54 (1H, s, H6'). ^13^C-NMR (75 MHz, CDCl_3_) δ 160.0, 153.0, 144.3, 142.8, 141.9, 133.3, 132.1, 130.1, 128.1, 122.0, 121.9. IR (ATR Ge, cm^−1^): 3332 (NH, CONH), 2360, 2342, 1690 (C=O, CONH), 1574, 1511, 1459, 1366, 1258, 1143, 1074, 1022, 901, 887, 669. Anal. Calcd. for C_11_H_5_Cl_4_N_3_O_1_ (MW 336.99): C, 39.21; H, 1.50; N, 12.47. Found: C, 39.49; H, 1.57; N, 12.42.

*5-Chloro-N-(3-bromophenyl)pyrazine-2-carboxamide* (**16**). White solid. Yield: 56%. mp 135.3–136.0 °C. ^1^H-NMR (500 MHz, CDCl_3_) δ 9.48 (1H, bs, NH), 9.26 (1H, d, *J* = 1.5 Hz, H3), 8.58 (1H, d, *J* = 1.5 Hz, H6), 7.99 (1H, t, *J* = 2.0 Hz, H2'), 7.65 (1H, d, *J* = 8.1 Hz, H6'), 7.32 (1H, d, *J* = 8.1 Hz, H4'), 7.26 (1H, t, *J* = 8.1 Hz, H5'). ^13^C-NMR (125 MHz, CDCl_3_) δ 159.8, 152.6, 144.3, 142.4, 142.2, 138.2, 130.5, 128.0, 122.8, 122.8, 118.3. IR (ATR Ge, cm^−1^): 3360 (NH, CONH), 1693 (C=O, CONH), 1586, 1521, 1455, 1421, 1273, 1251, 1135, 1024, 906, 788, 776, 681. Anal. Calcd. for C_11_H_7_Br_1_Cl_1_N_3_O_1_ (MW 312.55): C, 42.27; H, 2.26; N, 13.44. Found: C, 41.93; H, 2.07; N, 13.28.

*5-Chloro-N-(2-chloro-4-iodophenyl)pyrazine-2-carboxamide* (**18**). Pale beige solid. Yield: 55%. mp 175.6–177.3 °C. ^1^H-NMR (300 MHz, CDCl_3_) δ 10.13 (1H, bs, NH), 9.25 (1H, d, *J* = 1.2 Hz, H3), 8.62 (1H, d, *J* = 1.2 Hz, H6), 8.37 (1H, d, *J* = 8.8 Hz, H6'), 7.76 (1H, d, *J* = 1.8 Hz, H3'), 7.64 (1H, dd, *J* = 8.8 Hz, *J* = 1.8 Hz, H5'). ^13^C-NMR (75 MHz, CDCl_3_) δ 159.9, 152.8, 144.2, 142.7, 142.2, 137.3, 136.9, 133.9, 124.1, 122.4, 87.3. IR (ATR Ge, cm^−1^): 3326 (NH, CONH), 3079, 2360, 1687 (C=O, CONH), 1586, 1521, 1463, 1378, 1320, 1254, 1135, 1023, 901, 871, 832, 719, 705, 680. Anal. Calcd. for C_11_H_6_Cl_2_I_1_N_3_O_1_ (MW 394.00): C, 33.53; H, 1.54; N, 10.67. Found: C, 33.50; H, 1.49; N, 10.82.

*5-Chloro-N-(5-fluoro-2-methylphenyl)pyrazine-2-carboxamide* (**19**). White crystalline needles. Yield: 40%. mp 138.0–138.7 °C. ^1^H-NMR (300 MHz, CDCl_3_) δ 9.56 (1H, bs, NH), 9.26 (1H, d, *J* = 1.4 Hz, H3), 8.59 (1H, d, *J* = 1.4 Hz, H6), 8.12 (1H, dd, *J* = 11.0 Hz, *J* = 2.8 Hz, H6'), 7.15 (1H, dd, *J* = 8.2 Hz, *J* = 6.3 Hz, H3'), 6.80 (1H, dt, *J* = 8.2 Hz, *J* = 2.8 Hz, H4'), 2.35 (3H, s, CH_3_). ^13^C-NMR (75 MHz, CDCl_3_) δ 161.4 (d, *J* = 242.8 Hz), 159.6, 152.6, 144.2, 142.5, 136.1 (d, *J* = 11.2 Hz), 131.2 (d, *J* = 9.1 Hz), 122.6 (d, *J* = 3.1 Hz), 111.5 (d, *J* = 21.2 Hz), 108.3 (d, *J* = 27.2 Hz), 16.9. IR (ATR Ge, cm^−1^): 3364 (NH, CONH), 1696 (C=O, CONH), 1602, 1540, 1450, 1252, 1165, 1140, 1117, 1022, 899, 888, 818, 726, 662. Anal. Calcd. for C_12_H_9_Cl_1_F_1_N_3_O_1_ (MW 265.67): C, 54.25; H, 3.41; N, 15.82. Found: C, 54.37; H, 3.44; N, 15.99.

*5-Chloro-N-(2-chloro-5-methylphenyl)pyrazine-2-carboxamide* (**20**). White solid. Yield: 53%. mp 130.8–131.9 °C. ^1^H-NMR (300 MHz, CDCl_3_) δ 10.11 (1H, bs, NH), 9.25 (1H, d, *J* = 1.4 Hz, H3), 8.61 (1H, d, *J* = 1.4 Hz, H6), 8.44–8.38 (1H, m, H6'), 7.29 (1H, d, *J* = 8.2 Hz, H3'), 6.95–6.89 (1H, m, H4'), 2.38 (3H, s, CH_3_). ^13^C-NMR (75 MHz, CDCl_3_) δ 159.8, 152.5, 144.1, 142.6, 138.1, 133.5, 128.8, 126.1, 121.7, 120.5, 21.3. IR (ATR Ge, cm^−1^): 3319 (NH, CONH), 2360, 1689 (C=O, CONH), 1588, 1530, 1452, 1300, 1256, 1143, 1128, 1112, 1046, 1022, 895, 805, 690. Anal. Calcd. for C_12_H_9_Cl_2_N_3_O_1_ (MW 282.13): C, 51.09; H, 3.22; N, 14.89. Found: C, 50.85; H, 3.35; N, 14.94.

*5-Chloro-N-(5-chloro-2-hydroxyphenyl)pyrazine-2-carboxamide* (**21**). Pale yellow solid. Yield: 40%. mp 221.4–222.2 °C. ^1^H-NMR (300 MHz, DMSO-*d_6_*) δ 10.72 (1H, bs, NH), 10.05 (1H, s, OH), 9.11 (1H, d, *J* = 1.2 Hz, H3), 8.94 (1H, d, *J* = 1.2 Hz, H6), 8.33 (1H, d, *J* = 2.5 Hz, H6'), 7.02 (1H, dd, *J* = 8.7 Hz, *J* = 2.5 Hz, H4'), 6.93 (1H, d, *J* = 8.7 Hz, H3'). ^13^C-NMR (75 MHz, DMSO-*d_6_*) δ 159.8, 151.6, 145.8, 143.7, 143.4, 142.7, 126.9, 124.3, 122.8, 118.9, 116.1. IR (ATR Ge, cm^−1^): 3326 (NH, CONH), 3118, 2359, 2342, 1660 (C=O, CONH), 1543, 1453, 1426, 1257, 1196, 1148, 1122, 1020, 921, 875, 810, 740, 701, 652. Anal. Calcd. for C_11_H_7_Cl_2_N_3_O_2_ (MW 284.10): C, 46.51; H, 2.48; N, 14.79. Found: C, 46.42; H, 2.42; N, 14.68.

*5-Chloro-N-(3-chloro-4-hydroxyphenyl)pyrazine-2-carboxamide* (**22**). Pale yellow solid. Yield: 40%. mp 200.7–203.5 °C. ^1^H-NMR (300 MHz, DMSO-*d_6_*) δ 10.67 (1H, bs, NH), 10.04 (s, 1H, OH), 9.07 (1H, d, *J* = 1.4 Hz, H3), 8.89 (1H, d, *J* = 1.4 Hz, H6), 7.92 (1H, d, *J* = 2.2 Hz, H2'), 7.62 (1H, dd, *J* = 8.8 Hz, *J* = 2.2 Hz, H6'), 6.94 (1H, d, *J* = 8.8 Hz, H5'). ^13^C-NMR (75 MHz, DMSO-*d_6_*) δ 160.7, 151.0, 150.0, 144.1, 144.0, 143.1, 130.6, 122.4, 121.0, 119.2, 116.5. IR (ATR Ge, cm^−1^): 3343 (NH, CONH), 3233, 1761, 1649 (C=O, CONH), 1600, 1552, 1519, 1429, 1310, 1275, 1251, 1200, 1133, 1023, 895, 878, 828, 694, 665. Anal. Calcd. for C_11_H_7_Cl_2_N_3_O_2_ (MW 284.10): C, 46.51; H, 2.48; N, 14.79. Found: C, 46.69; H, 2.57; N, 14.61.

*5-Chloro-N-(2-hydroxy-5-nitrophenyl)pyrazine-2-carboxamide* (**23**). Pale yellow solid. Yield: 42%. mp 235.0–236.3 °C. ^1^H-NMR (300 MHz, DMSO-*d_6_*) δ 10.07 (1H, bs, NH), 9.17–9.13 (1H, m, H6'), 9.11–9.09 (1H, m, H3), 8.97–8.92 (1H, m, H6), 7.93 (1H, dd, *J* = 9.1 Hz, *J* = 2.8 Hz, H4'), 7.06 (1H, d, *J* = 9.1 Hz, H3'). ^13^C-NMR (75 MHz, DMSO-*d_6_*) δ 160.2, 153.3, 151.8, 143.8, 143.5, 142.4, 139.5, 125.9, 121.4, 114.6. IR (ATR Ge, cm^−1^): 3338 (NH, CONH), 3091, 2360, 2342, 1663 (C=O, CONH), 1593, 1544, 1520, 1497, 1456, 1337, 1289, 1262, 1149, 1117, 1074, 1022, 905, 746, 736, 703. Anal. Calcd. for C_11_H_7_Cl_1_N_4_O_4_ (MW 294.65): C, 44.84; H, 2.39; N, 19.02. Found: C, 44.59; H, 2.62; N, 19.12.

*5-Chloro-N-(2-nitrophenyl)pyrazine-2-carboxamide* (**24**). Yellow solid. Yield: 33%. mp 156.3–156.8 °C. ^1^H-NMR (300 MHz, CDCl_3_) δ 12.36 (1H, bs, NH), 9.19 (1H, d, *J* = 1.4 Hz, H3), 8.90 (1H, dd, *J* = 8.5 Hz, *J* = 1.5 Hz, H3'), 8.62 (1H, d, *J* = 1.4 Hz, H6), 8.21 (1H, dd, *J* = 8.5 Hz, *J* = 1.5 Hz, H6'), 7.70–7.62 (1H, m, H4'), 7.25–7.16 (1H, m, H5'). ^13^C-NMR (75 MHz, CDCl_3_) δ 161.2, 152.9, 144.4, 143.0, 142.3, 137.0, 136.0, 133.9, 126.0, 124.1, 122.1. IR (ATR Ge, cm^−1^): 3308 (NH, CONH), 1687 (C=O, CONH), 1610, 1584, 1502, 1428, 1340, 1312, 1275, 1256, 1139, 1022, 906, 862, 731, 688. Anal. Calcd. for C_11_H_7_Cl_1_N_4_O_3_ (MW 278.65): C, 47.41; H, 2.53; N, 20.11. Found: C, 47.13; H, 2.57; N, 20.31.

*5-Chloro-N-(3-cyanophenyl)pyrazine-2-carboxamide* (**26**). Pale beige solid. Yield: 45%. mp 206.8–208.3 °C. ^1^H-NMR (300 MHz, DMSO-*d_6_*) δ 11.1 (1H, bs, NH), 9.11 (1H, s, H3), 8.93 (1H, s, H6), 8.32 (1H, s, H2'), 8.23–8.14 (1H, m, H6'), 7.62–7.55 (2H, m, H4', H5'). ^13^C-NMR (75 MHz, DMSO-*d_6_*) δ 161.6, 151.4, 144.4, 143.5, 143.2, 139.2, 130.3, 128.0, 125.5, 123.6, 118.8, 111.7. IR (ATR Ge, cm^−1^): 3304 (NH, CONH), 3073, 2360, 2241 (CN, nitrile), 1683 (C=O, CONH), 1589, 1552, 1437, 1300, 1254, 1134, 1023, 894, 882, 797, 789, 679. Anal. Calcd. for C_12_H_7_Cl_1_N_4_O_1_ (MW 258.66): C, 55.72; H, 2.73; N, 21.66. Found: C, 55.83; H, 2.81; N, 21.52.

*5-Chloro-N-(4-cyanophenyl)pyrazine-2-carboxamide* (**27**). White solid. Yield: 42%. mp 225.4–226.8 °C. ^1^H-NMR (300 MHz, DMSO-*d_6_*) δ 11.15 (1H, bs, NH), 9.11 (1H, d, *J* = 1.4 Hz, H3), 8.93 (1H, d, *J* = 1.4 Hz, H6), 8.14–8.07 (2H, m, AA', BB', H3', H5'), 7.86–7.79 (2H, m, AA', BB', H2', H6'). ^13^C-NMR (75 MHz, DMSO-*d_6_*) δ 161.8, 151.4, 144.5, 143.5, 143.2, 142.6, 133.3, 120.9, 119.1, 106.3. IR (ATR Ge, cm^−1^): 3348 (NH, CONH), 2360, 2228 (CN, nitrile), 1700 (C=O, CONH), 1587, 1518, 1455, 1409, 1313, 1245, 1173, 1132, 1023, 897, 862, 823, 665. Anal. Calcd. for C_12_H_7_Cl_1_N_4_O_1_ (MW 258.66): C, 55.72; H, 2.73; N, 21.66. Found: C, 55.49; H, 2.67; N, 21.71.

*5-Chloro-N-(3-(trifluoromethyl)phenyl)pyrazine-2-carboxamide* (**28**). White solid. Yield: 71%. mp 121.5–122.5 °C. ^1^H-NMR (300 MHz, CDCl_3_) δ 9.60 (1H, bs, NH), 9.27 (1H, d, *J* = 1.4 Hz, H3), 8.59 (1H, d, *J* = 1.4 Hz, H6), 8.03 (1H, bs, H2'), 7.95 (1H, d, *J* = 8.0 Hz, H4'), 7.52 (1H, t, *J* = 8.0 Hz, H5'), 7.44 (1H, d, *J* = 8.0 Hz, H6'). ^13^C-NMR (75 MHz, CDCl_3_) δ 160.1, 152.8, 144.3, 142.5, 142.1, 137.5, 131.7 (q, *J* = 32.4 Hz), 129.8, 123.7 (q, *J* = 272.6 Hz), 122.9, 121.6 (q, *J* = 3.7 Hz), 116.6 (q, *J* = 4.0 Hz). IR (ATR Ge, cm^−1^): 3367 (NH, CONH), 2359, 1686 (C=O, CONH), 1608, 1544, 1453, 1340, 1325, 1136, 1119, 1071, 1024, 918, 903, 802, 699, 660. Anal. Calcd. for C_12_H_7_Cl_1_F_3_N_3_O_1_ (MW 301.65): C, 47.78; H, 2.34; N, 13.93. Found: C, 47.95; H, 2.42; N, 13.80.

*5-Chloro-N-(4-(trifluoromethyl)phenyl)pyrazine-2-carboxamide* (**29**). White solid. Yield: 78%. mp 179.8–181.1 °C. ^1^H-NMR (300 MHz, CDCl_3_) δ 9.63 (1H, bs, NH), 9.28 (1H, d, *J* = 1.2 Hz, H3), 8.59 (1H, d, *J* = 1.2 Hz, H6), 7.90–7.84 (2H, m, AA', BB', H3', H5'), 7.69–7.62 (2H, m, AA', BB', H2', H6'). ^13^C-NMR (75 MHz, CDCl_3_) δ 160.1, 152.8, 144.3, 142.4, 142.1, 140.0, 126.8 (q, *J* = 32.9 Hz), 126.5 (q, *J* = 4.0 Hz), 123.9 (q, *J* = 271.7 Hz), 119.5. IR (ATR Ge, cm^−1^): 3372 (NH, CONH), 2359, 1690 (C=O, CONH), 1530, 1412, 1322, 1315, 1162, 1143, 1119, 1066, 1028, 902, 846, 666. Anal. Calcd. for C_12_H_7_Cl_1_F_3_N_3_O_1_ (MW 301.65): C, 47.78; H, 2.34; N, 13.93. Found: C, 47.58; H, 2.28; N, 13.71.

*4-(5-Chloropyrazine-2-carboxamido)-2-hydroxybenzoic acid* (**30**). Pale yellow solid. Yield: 18%. mp 240.0–244.0 dec °C. ^1^H-NMR (300 MHz, DMSO-*d_6_*) δ 10.62 (1H, bs, NH), 9.09 (1H, d, *J* = 1.1 Hz, H3), 8.91 (1H, d, *J* = 1.1 Hz, H6), 7.66 (1H, d, *J* = 8.5 Hz, H5'), 7.36 (1H, d, *J* = 2.1 Hz, H2'), 7.21 (1H, dd, *J* = 8.5 Hz, *J* = 2.1 Hz, H6'). ^13^C-NMR (75 MHz, DMSO-*d_6_*) δ 171.9, 163.0, 161.2, 151.1, 145.4, 144.3, 144.1, 143.1, 130.6, 109.6, 107.6. IR (ATR Ge, cm^−1^): 3389 (NH, CONH), 1694 (C=O, CONH), 1600, 1133, 1024, 785. Anal. Calcd. for C_12_H_8_Cl_1_N_3_O_4_ (MW 293.66): C, 49.08; H, 2.75; N, 14.31. Found: C, 48.86; H, 2.79; N, 14.17.

### 3.4. Determination of Lipophilicity by HPLC (Logk)

*Instrumentation:* Agilent Technologies 1200 SL liquid chromatograph with Diode-array Detector SL G1315C (Agilent Technologies Inc., Colorado Springs, CO, USA); pre-column ZORBAX XDB-C18 5 µm, 4 × 4 mm, Part No. 7995118-504 (Agilent Technologies Inc.) and column ZORBAX Eclipse XDB-C18 5 µm, 4.6 × 250 mm, Part No. 7995118-585 (Agilent Technologies Inc.). The separation process was controlled by Agilent ChemStation, version B.04.02 extended by spectral module (Agilent Technologies Inc.). Mobile phase consisted of MeOH (HPLC grade, 70%) and H_2_O (HPLC-Milli-Q Grade, 30%).

*Conditions*: Flow rate 1.0 mL/min, sample injection volume 20 µL, column temperature 30 °C, detection wavelength 210 nm, monitor wavelength 270 nm. Retention times (t_R_) were measured in minutes. The dead time of the system (t_D_) was determined as the retention time of KI methanol solution. Capacity factors k for individual compounds were calculated according to the formula k = (t_R_ − t_D_)/t_D_. Log*k*, calculated from the capacity factor *k*, is used as the lipophilicity index converted to log scale.

### 3.5. Biological Methods

#### 3.5.1. Evaluation of *In Vitro* Antimycobacterial Activity

Microdilution panel method. Tested strains *M. tuberculosis* H37Rv CNCTC My 331/88, *M. kansasii* Hauduroy CNCTC My 235/80, *M. avium* ssp. *avium* Chester CNCTC My 80/72 and *M. avium* CNCTC My 152/73 were obtained from Czech National Collection of Type Cultures (CNCTC), National Institute of Public Health, Prague, Czech Republic. Tested compounds were dissolved and diluted in DMSO and mixed with growth media (Šula's semisynthetic medium, pH = 5.6, Trios, Prague, Czech Republic) to final concentrations of 100–50–25–12.5–6.25–3.13–1.56 μg/mL. The mycobacterial suspensions for each strain were prepared by dilution of the basic isotonic saline suspension (McFarland 0.5–1.0) by 10^−1^ and 10^−3^. These suspensions were used to inoculate the testing wells so each compound was tested in duplicates at two different concentrations of mycobacterial suspension. The assay involved positive controls of mycobacterial growth (DMSO plus broth). Pyrazinamide (PZA) and isoniazid (INH) were used as standards. The testing plates were incubated at 36 ± 1 °C until the growth of mycobacteria was visually evident in positive control wells (usually 10–14 days). The MIC (μg/mL) was determined visually as the lowest concentration of tested compound that inhibited the growth of mycobacteria. The difference in MIC of a compound read from the parallel lines with different concentrations of mycobacterial suspension must not exceed one step on the dilution scale.

#### 3.5.2. HepG2 Cytotoxicity Determination

The human hepatocellular liver carcinoma cell line HepG2 (p 26–27, p 32–33) purchased from Health Protection Agency Culture Collections (ECACC, Salisbury, UK) was routinely cultured in MEM (Minimum Essentials Eagle Medium) (Sigma-Aldrich) supplemented with 10% foetal bovine serum (PAA), 1% L-Glutamine solution (Sigma-Aldrich) and non-essential amino acid solution (Sigma-Aldrich) in a humidified atmosphere containing 5% CO_2_ at 37 °C. For subculturing, the cells were harvested after trypsin/EDTA (Sigma-Aldrich) treatment at 37 °C. The cells treated with the tested substances were used as experimental groups. Untreated HepG2 cells were used as control groups. The cells were seeded in density 1 × 10^4^ cells per well in a 96-well plate. Next day, the cells were treated with each of the tested substances dissolved in DMSO by dilution so that a final solution contained less than 1% of DMSO in the medium. The tested compounds were prepared in incubation concentrations 0–100 µM in triplicates. The controls: 100% cell viability, 0% cell viability (the cells treated with 10% DMSO), no cell control and vehiculum controls were also prepared in triplicates. After 24 h of incubation in a humidified atmosphere containing 5% CO_2_ at 37 °C, the reagent from the kit CellTiter 96 AQueous One Solution Cell Proliferation Assay (CellTiter 96; Promega) was added. After 2 h incubation at 37 °C the absorbance was recorded at 490 nm. A standard toxicological parameter IC_50_ was calculated in each of the tested substances using GraphPad Prism software (version 5.02).

#### 3.5.3. CHO-K1 and ACHN Cytotoxicity Determination

The standard MTT assay (Sigma Aldrich) was applied according to the manufacturer’s protocol on renal cell adenocarcinoma (ACHN) and Chinese hamster ovary (CHO-K1) cell lines (all from ECACC, Salisbury, UK). The cells were cultured according to ECACC recommended conditions and seeded in a density of 8 × 10^3^, 12 × 10^3^ per well respectively for CHO-K1, ACHN cells. Cells were exposed for tested compounds for 24 h, then the medium was replaced for a medium containing 10 μM of MTT and cells were allowed to produce formazan for another approximately 1 h under surveillance. Then, medium with MTT was sucked out and crystals of formazan were dissolved in DMSO. Cell viability was assessed spectrophotometrically by the amount of formazan produced. Absorbance was measured at 570 nm with 650 nm reference wavelength on Synergy HT (BioTek, Winooski, VT, USA). IC_50_ was then calculated from the triplicates using non-linear regression (four parameters) of GraphPad Prism 5 software. Final IC_50_ value was obtained as a mean of at least three independent measurements.

#### 3.5.4. Evaluation of *In Vitro* Antibacterial Activity

Microdilution broth method [21]. The organisms examined included strains from Czech Collection of Microorganisms (Brno, Czech Republic): *Staphylococcus aureus* CCM 4516/08, *Escherichia coli* CCM 4517, *Pseudomonas aeruginosa* CCM 1961. These strains are recommended as standards for testing of antibacterial activities. Other strains were clinical isolates (Department of Clinical Microbiology, University Hospital and Faculty of Medicine in Hradec Králové, Charles University in Prague, Czech Republic): *Staphylococcus aureus* H 5996/08-methicilin resistant (MRSA), *Staphylococcus epidermidis* H 6966/08, *Enterococcus* sp. J 14365/08, *Klebsiella pneumoniae* D 11750/08, *Klebsiella pneumoniae* J 14368/08-ESBL positive. All strains were subcultured on Mueller-Hinton agar (MHA) (Difco/Becton Dickinson, Detroit, MI, USA) at 35 °C and maintained on the same medium at 4 °C. Prior to testing, each strain was passaged onto MHA. Bacterial inocula were prepared by suspending in sterile 0.85% saline. The cell density of the inoculum was adjusted to yield suspension of density equivalent 0.5 McFarland scale (1.5 × 10^8^ viable CFU/mL). The compounds were dissolved in DMSO, and the antibacterial activity was determined in Mueller-Hinton liquid broth (Difco/Becton Dickinson, Detroit, MI, USA), buffered to pH 7.0. Controls consisted of medium and DMSO alone. The final concentration of DMSO in the test medium did not exceed 1% (v/v) of the total solution composition. The minimum inhibitory concentration (MIC), defined as 95% inhibition of bacterial growth as compared to control, was determined after 24 and 48 h of static incubation at 35 °C.

#### 3.5.5. Evaluation of *In Vitro* Antifungal Activity

The Department of Medical and Biological Sciences at the Faculty of Pharmacy in Hradec Králové, Charles University in Prague, Czech Republic, performed the antifungal susceptibility assays, which was carried out using microdilution broth method [22]. Compounds were dissolved in DMSO and diluted in a twofold manner with RPMI 1640 medium with glutamine buffered to pH 7.0 (3-morpholinopropane-1-sulfonic acid). The final concentration of DMSO in the tested medium did not exceed 2.5% (v/v) of the total solution composition. Static incubation was performed in the dark and humid, at 35 °C for 24 and 48 h (respectively 72 and 120 h for *Trichophyton mentagrophytes*). Drug-free controls were included. Fluconazole was used as standard. Tested species: *Candida albicans* ATCC 44859, *C. tropicalis* 156, *C. krusei* E28, *C. glabrata* 20/I, *Trichosporon asahii* 1188, *Aspergillus fumigates* 231, *Absidia corymbifera* 272 and *Trichophyton mentagrophytes* 445.

## 4. Conclusions

To conclude, we have successfully demonstrated that the anilides of 5-Cl-POA (5-chloro-*N-*phenylpyrazine-2-carboxamides) possess significant antimycobacterial activity, especially against *M. tuberculosis* H37Rv. We determined the basic structure-activity and structure-toxicity relationships. The phenyl part tolerated many various substituents while maintaining the activity. Most of the compounds exerted significant *in vitro* hepatotoxicity as evaluated by HepG2 cell line model—however, hydroxyl and other hydrophilic substituents decreased the cytotoxicity. 5-Chloro-*N*-(5-chloro-2-hydroxyphenyl)pyrazine-2-carboxamide (**21**) possessed the broadest spectrum of antimycobacterial activity and inhibited all of the tested strains (including the strains resistant to PZA), and at the same time had one of the lowest HepG2 cytotoxicity. 4-(5-Chloropyrazine-2-carboxamido)-2-hydroxybenzoic acid (**30**), although not the absolutely most active in the presented series, was approximately 5–10 times more active in comparison with first-line antitubercular agent PZA (MIC for *M. tbc* H37Rv—10 µM for **30**, 51–102 µM for PZA). Notably, compound **30** was rated as non-toxic in CHO-K1 and ACHN cell line models. Therefore, compounds **21** (for its broad spectrum of activity) and **30** (for its non-toxicity) can be highlighted as the lead structures for further development. The 5-chloro substituent on the pyrazine core will allow easy structural modifications *via* nucleophilic substitution.

## Supplementary Materials

Supplementary materials can be accessed at: http://www.mdpi.com/1420-3049/18/12/14807/s1.

## References

[1] World Health Organization (2013). Global Tuberculosis Report 2013.

[2] Cynamon M.H., Speirs R.J., Welch J.T. (1998). *In vitro* antimycobacterial activity of 5-chloropyrazinamide. Antimicrob. Agents Chemother..

[3] Zimhony O., Cox J.S., Welch J.T., Vilcheze C., Jacobs W.R. (2000). Pyrazinamide inhibits the eukaryotic-like fatty acid synthetase I (FASI) of *Mycobacterium tuberculosis*. Nat. Med..

[4] Boshoff H.I., Mizrahi V., Barry C.E. (2002). Effects of pyrazinamide on fatty acid synthesis by whole mycobacterial cells and purified fatty acid synthase I. J. Bacteriol..

[5] Ngo S.C., Zimhony O., Chung W.J., Sayahi H., Jacobs W.R., Welch J.T. (2007). Inhibition of isolated mycobacterium tuberculosis fatty acid synthase I by pyrazinamide analogs. Antimicrob. Agents Chemother..

[6] Sayahi H., Pugliese K.M., Zimhony O., Jacobs W.R., Shekhtman A., Welch J.T. (2012). Analogs of the antituberculous agent pyrazinamide are competitive inhibitors of NADPH binding to *M. tuberculosis* fatty acid synthase I. Chem. Biodivers..

[7] Ahmad Z., Tyagi S., Minkowski A., Almeida D., Nuermberger E.L., Peck K.M., Welch J.T., Baughn A.S., Jacobs W.R., Grosset J.H. (2012). Activity of 5-chloropyrazinamide in mice infected with *Mycobacterium tuberculosis* or *Mycobacterium bovis*. Indian J. Med. Res..

[8] Zhang Y., Mitchison D. (2003). The curious characteristics of pyrazinamide: A review. Int. J. Tuberc. Lung Dis..

[9] Dolezal M., Kesetovic D., Zitko J. (2011). Antimycobacterial evaluation of pyrazinoic acid reversible derivatives. Curr. Pharm. Des..

[10] Dolezal M., Zitko J., Jampilek J., Cardona P.-J. (2012). Pyrazinecarboxylic Acid Derivatives with Antimycobacterial Activity. Understanding Tuberculosis—New Approaches to Fighting Against Drug Resistance.

[11] Servusova B., Vobickova J., Paterova P., Kubicek V., Kunes J., Dolezal M., Zitko J. (2013). Synthesis and antimycobacterial evaluation of *N*-substituted 5-chloropyrazine-2-carboxamides. Bioorg. Med. Chem. Lett..

[12] Wardell S., de Souza M.V.N., Vasconcelos T.R.A., Ferreira M.D.L., Wardell J.L., Low J.N., Glidewell C. (2008). Patterns of hydrogen bonding in mono- and disubstituted *N*-arylpyrazinecarboxamides. Acta Crystallogr. Sect. B Struct. Sci..

[13] Zitko J., Paterova P., Kubicek V., Mandikova J., Trejtnar F., Kunes J., Dolezal M. (2013). Synthesis and antimycobacterial evaluation of pyrazinamide derivatives with benzylamino substitution. Bioorg. Med. Chem. Lett..

[14] Tostmann A., Boeree M.J., Peters W.H.M., Roelofs H.M.J., Aarnoutse R.E., van der Ven A., Dekhuijzen P.N.R. (2008). Isoniazid and its toxic metabolite hydrazine induce *in vitro* pyrazinamide toxicity. Int. J. Antimicrob. Agents.

[15] Bispo M.D.F., Goncalves R.S.B., Lima C.H.D., Cardoso L.N.D., Lourenco M.C.S., de Souza M.V.N. (2012). Synthesis and Antitubercular Evaluation of *N*-Arylpyrazine and *N,N’*-Alkyl-diylpyrazine-2-carboxamide Derivatives. J. Heterocycl. Chem..

[16] Servusova B., Eibinova D., Dolezal M., Kubicek V., Paterova P., Pesko M., Kral’ova K. (2012). Substituted N-Benzylpyrazine-2-carboxamides: Synthesis and Biological Evaluation. Molecules.

[17] Tostmann A., Boeree M.J., Aarnoutse R.E., de Lange W.C.M., van der Ven A., Dekhuijzen R. (2008). Antituberculosis drug-induced hepatotoxicity: Concise up-to-date review. J. Gastroenterol. Hepatol..

[18] Singh M., Sasi P., Rai G., Gupta V.H., Amarapurkar D., Wangikar P.P. (2011). Studies on toxicity of antitubercular drugs namely isoniazid, rifampicin, and pyrazinamide in an *in vitro* model of HepG2 cell line. Med. Chem. Res..

[19] Singh M., Sasi P., Gupta V.H., Rai G., Amarapurkar D.N., Wangikar P.P. (2012). Protective effect of curcumin, silymarin and *N*-acetylcysteine on antitubercular drug-induced hepatotoxicity assessed in an *in vitro* model. Hum. Exp. Toxicol..

[20] Owen T.C. (1993). Tetrazolium Compounds for Cell Viability Assays. U.S. Patent.

[21] Jones R.N., Barry A.L. (1987). Optimal dilution susceptibility testing conditions, recommendations for MIC interpretation, and quality control guidelines for the ampicillin-sulbactam combination. J. Clin. Microbiol..

[22] National Committee for Clinical Laboratory Standards (2004). Method for Antifungal Disc Diffusion Susceptibility Testing of Yeasts: Approved Guideline M44-A.

