# Supplementary material for: Synthesis, Antimycobacterial Activity and In Vitro Cytotoxicity of 5-Chloro-N-phenylpyrazine-2-carboxamides
Source: Molecules. 2013 Dec 2;18(12):14807–25. doi: 10.3390/molecules181214807 (PMC6270209; doi:10.3390/molecules181214807)
Supplement: Supplementary file 1 [file molecules-18-14807-s001.pdf]

Supplementary Materials

^{1}H-, ^{13}C-NMR and mass spectra of Compound **1** (internal laboratory code JZ-90).

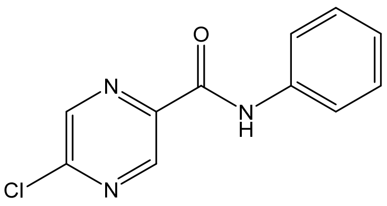

Chemical Formula: $C_{11}H_8ClN_3O$
Molecular Weight: 233,65

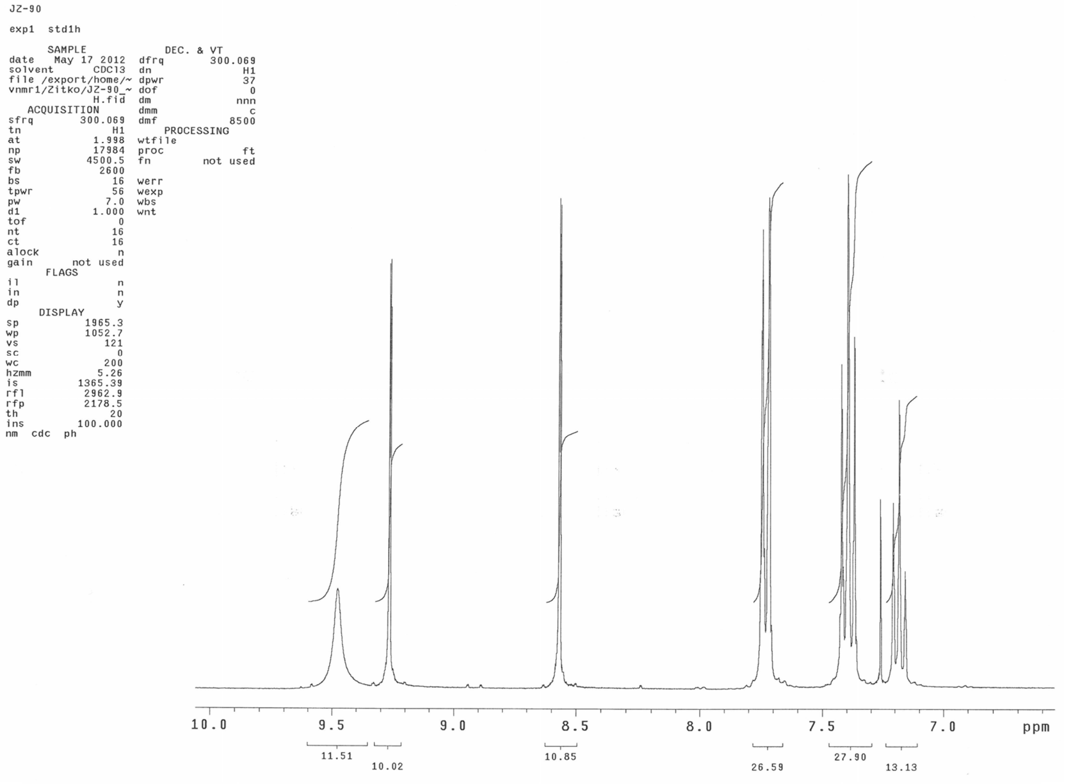

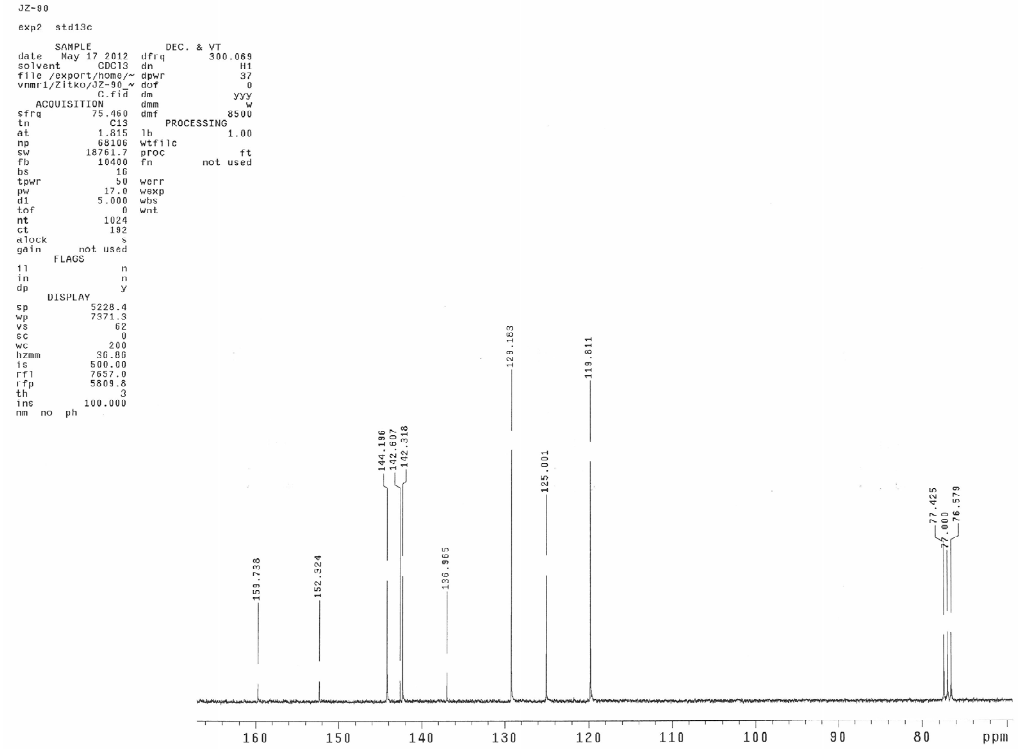

JZ-90 MS1

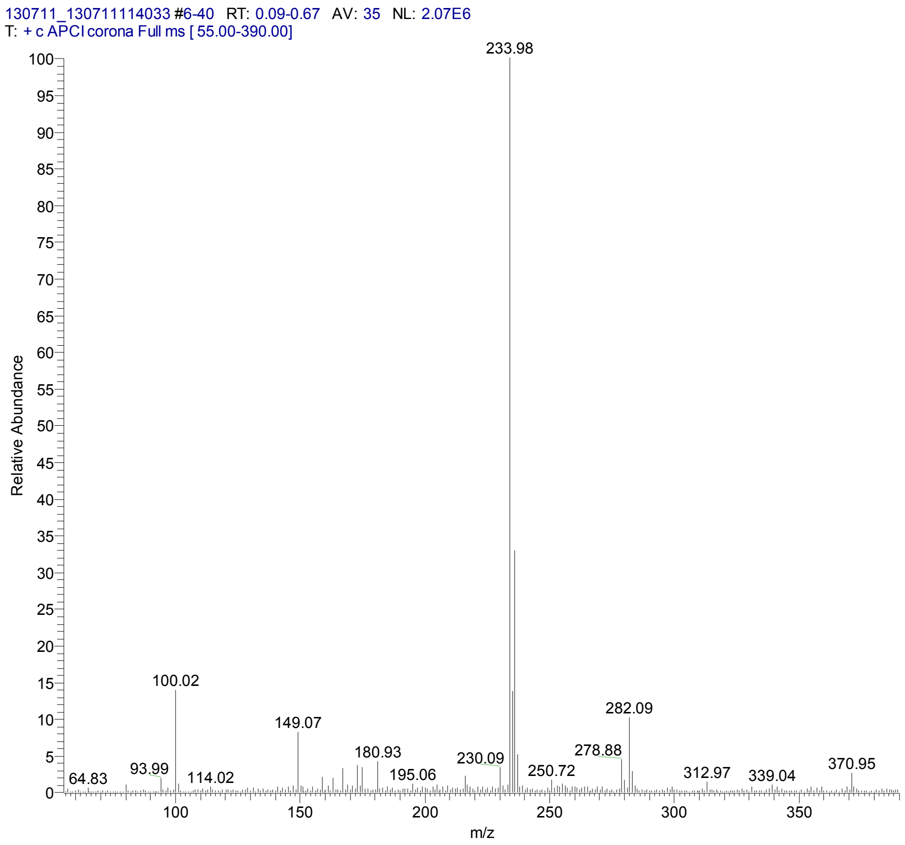

MS2_234

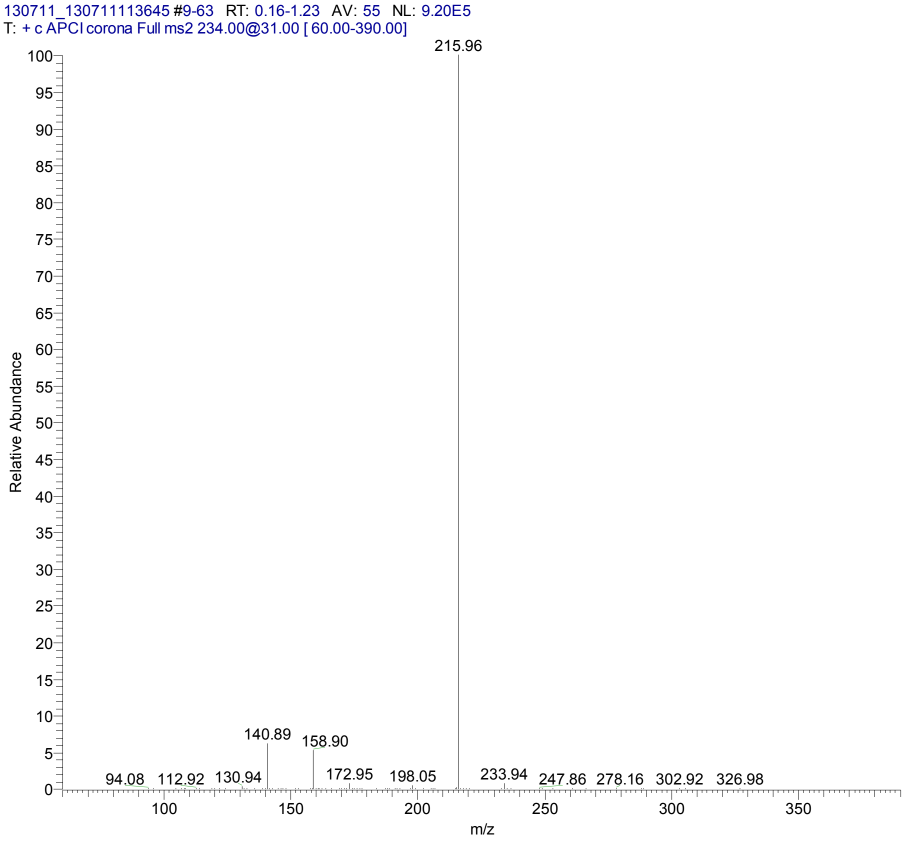

MS3_216

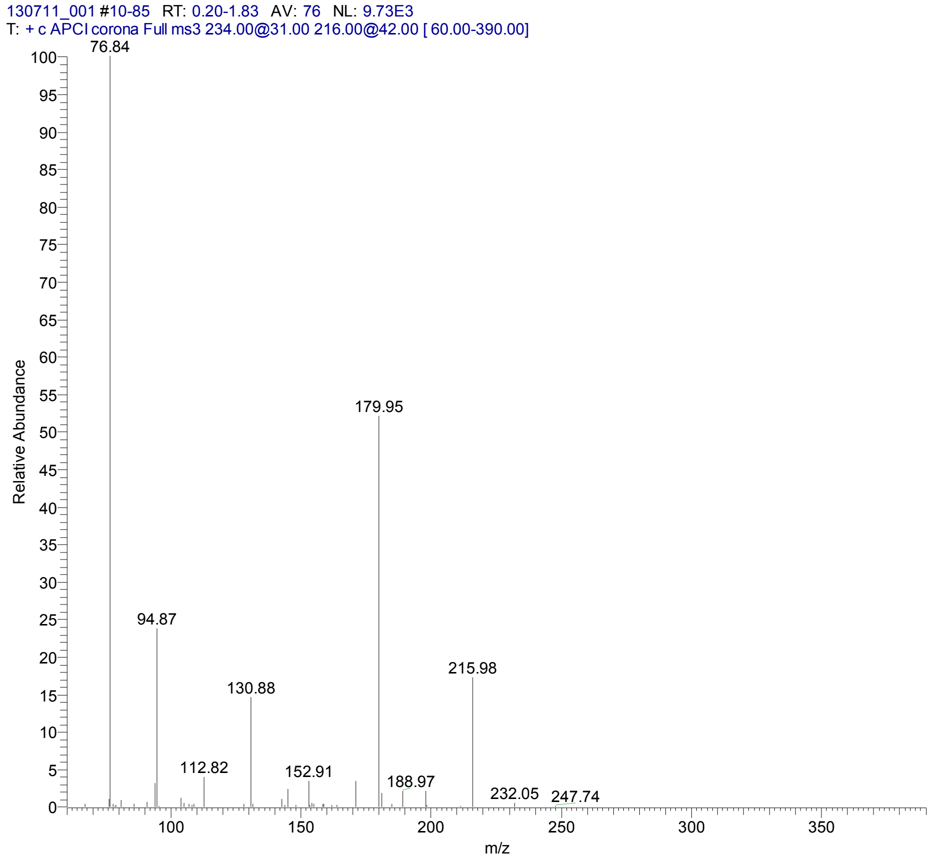

MS2_236

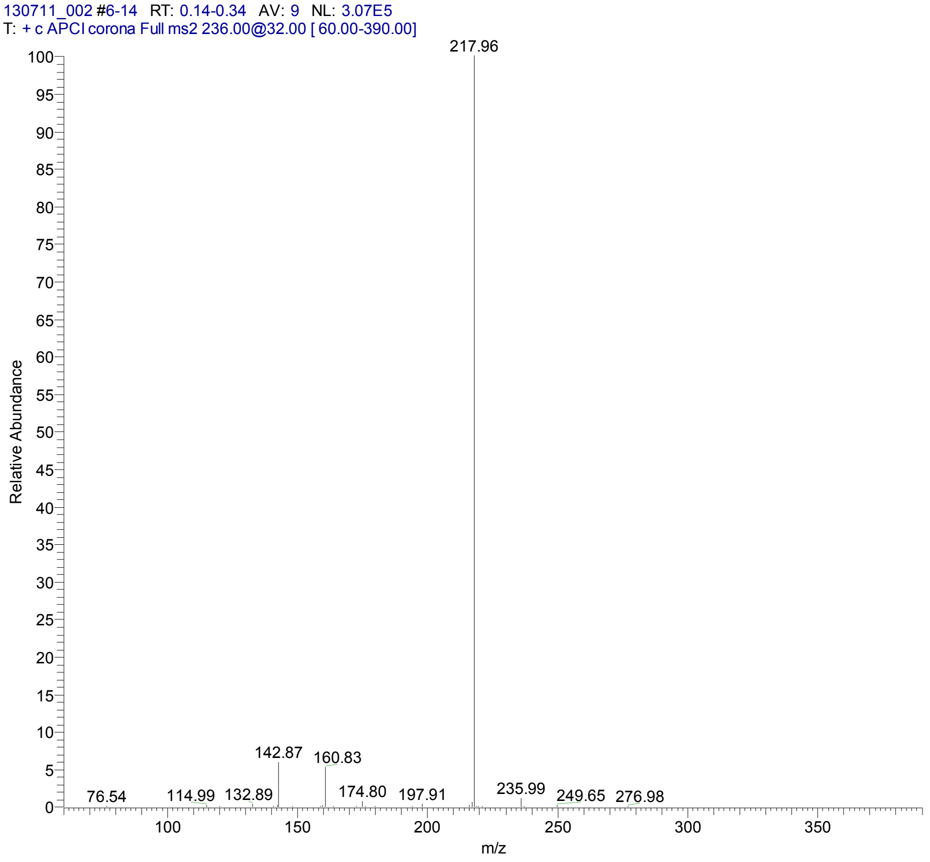

MS3_218

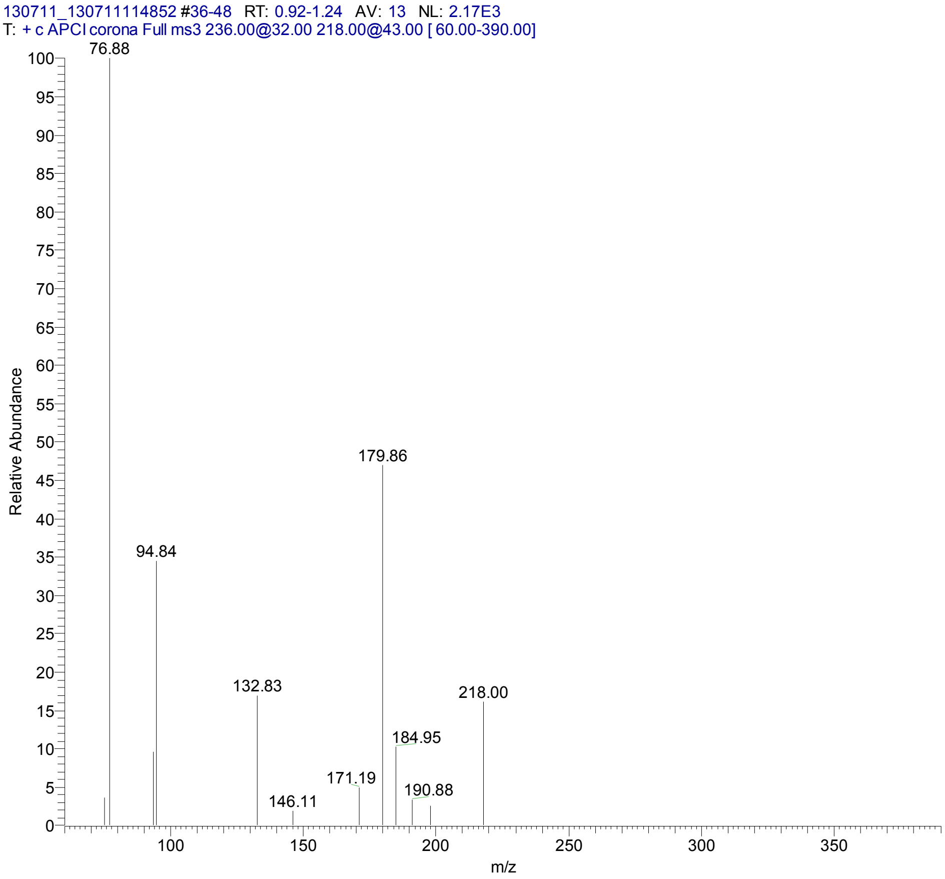
130711_130711114852 #36-48 RT: 0.92-1.24 AV: 13 NL: 2.17E3
T: + c APCI corona Full ms3 236.00@32.00 218.00@43.00 [60.00-390.00]
Relative Abundance
m/z
76.88
94.84
132.83
146.11
171.19
179.86
184.95
190.88
218.00
